# A Highly Responsive Silicon Nanowire/Amplifier MOSFET Hybrid Biosensor

**DOI:** 10.1038/srep12286

**Published:** 2015-07-21

**Authors:** Jieun Lee, Jaeman Jang, Bongsik Choi, Jinsu Yoon, Jee-Yeon Kim, Yang-Kyu Choi, Dong Myong Kim, Dae Hwan Kim, Sung-Jin Choi

**Affiliations:** 1 School of Electrical Engineering, Kookmin University, Seoul 136-702, Republic of Korea; 2 Department of Electrical Engineering, Yale University, New Haven, Connecticut 06511, United States; 3 Department of Electrical Engineering, Korea Advanced Institute of Science and Technology, Daejeon 305-701, Korea

## Abstract

This study demonstrates a hybrid biosensor comprised of a silicon nanowire (SiNW) integrated with an amplifier MOSFET to improve the current response of field-effect-transistor (FET)-based biosensors. The hybrid biosensor is fabricated using conventional CMOS technology, which has the potential advantage of high density and low noise performance. The biosensor shows a current response of 5.74 decades per pH for pH detection, which is 2.5 × 10^5^ times larger than that of a single SiNW sensor. In addition, we demonstrate charged polymer detection using the biosensor, with a high current change of 4.5 × 10^5^ with a 500 nM concentration of poly(allylamine hydrochloride). In addition, we demonstrate a wide dynamic range can be obtained by adjusting the liquid gate voltage. We expect that this biosensor will be advantageous and practical for biosensor applications which requires lower noise, high speed, and high density.

Highly sensitive biological detection is required for disease diagnosis^1^, drug discovery^2^, and biomolecular analysis^3^. The utilization of field-effect transistor (FET)-based sensor structures^1^,^4^,^5^,^6^,^7^ has been one of the most promising solutions for label-free real-time detection compared with other biosensors, including surface plasmon resonance^8^, microcantilevers^9^, and an array of fluorescence sensors^10^. FET-based silicon nanowire (SiNW) biosensors have great potential due to advantages which include direct electrical readouts, high sensitivity, and the potential to integrate them with complementary metal-oxide semiconductor (CMOS) circuits^11^,^12^,^13^,^14^,^15^.

SiNW biosensors operate by the change of current flowing through the SiNW channel which is induced by the change in surface charges, such as from a biomolecule binding reaction^16^. However, this current change often needs to be amplified, and in some applications current drive is important. Thus, the “current response”, which we define as the log of I/I_0_ per pH; (*i.e*. how many decades of current change per pH change), where I is the modified current and I_0_ is the initial current value, is an important metric. Many research groups have focused on efforts to improve current response. One of the most widely used methods is to reduce the doping concentration and nanowire width^17^,^18^. However, it is difficult to decrease the doping concentration of Si to below 10^15^ cm^−3^. In addition, low fabrication yield and low reproducibility can be expected when decreasing the device width. Another way to increase current response is to control the operating bias point of the biosensor by tuning gate bias. The operation of the biosensor in the subthreshold region exhibits the highest current response (although with an increase in noise^19^), as has already been shown theoretically and experimentally^20^,^21^.

In the subthreshold region of FETs, the subthreshold swing (SS), *i.e*., the inverse of the subthreshold slope, is defined as the gate voltage (V_G_) change needed to cause a one-order-of-magnitude change in the drain current and is expressed as


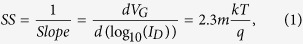


where *k* is the Boltzmann constant, *T* is the absolute temperature, *q* is the electric charge, and *m* is the ideality factor^22^. The minimum value of SS at room temperature can be achieved at 59.6 mV/decade when the minimum value of *m* is 1.

For pH sensors, which are the basis of broad sensor applications, the change in the surface potential of SiO_2_ with changing pH levels depends on the density and activation of surface sites, the buffer ionic strength, and the composition. The maximum change in the potential per pH is defined by the Nernst limit associated with an electrolyte and a site-binding surface, which is 59.6 mV/pH at room temperature^23^. Therefore, in the ideal case, the maximum ratio of current change (I/I_0_) per pH can intrinsically reach 10 (*i.e.*, 1 decade/pH) in the subthreshold region. However, because of sensor surface protonation affinity, the current change per pH with a SiO_2_ surface is always lower than the expected value^23^. In this paper, we demonstrate a new type of hybrid biosensor that combines the SiNW and conventional MOSFET, serving as an amplifier, to exceed the aforementioned current response intrinsic limit of 1 decade/pH. We demonstrate that the current response of the hybrid biosensor can be amplified considerably beyond the current response limit of the single SiNW biosensor. In addition, the hybrid biosensor is monolithically integrated on the same wafer using conventional top-down CMOS technology, indicating that it will be able to have the lower noise performance of amplification compared with laboratory instruments.

A schematic of the hybrid SiNW-MOSFET biosensor is shown in Fig. 1a. In this configuration, the SiNW device plays the role of a sensor and is exposed to the solution, whereas the MOSFET acts as a transducer to amplify the current response and is insulated from the solution. The SiNW and MOSFET are monolithically integrated and electrically connected by a metal line using conventional CMOS technology. The gate of the MOSFET and the drain of the SiNW FET are connected and are driven by the current source (I_IN_). Therefore, the drain voltage of the SiNW FET can be varied according to the conductance of the SiNW, which can control the gate voltage (V_G_) of the MOSFET. Figure 1b shows scanning electron microscope (SEM) images of the fabricated hybrid SiNW-MOSFET biosensor. The passivation layer around the SiNW channel region was removed to enable sensing, whereas regions outside the SiNW channel are passivated with a SiO_2_ layer to prevent leakage current through solution. The sensing area is located at the center of the chip, and all metal lines are extended to the edge of the chip to isolate the probe tips from the solution (Fig. 1c). Details of the fabrication process are discussed in Supplementary Figure S1 online.

## Results and Discussion

**Figure 2:**
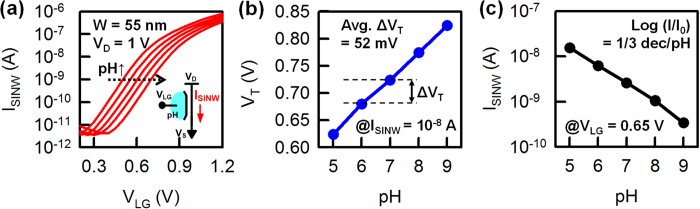
(**a**) Transfer characteristics of an n-type single SiNW FET as a pH sensor with a sweeping V_LG_. The SiNW FET was functionalized with APTES to apply amine (-NH_2_) groups to the sensor surface. The SS of the SiNW FET is 147 mV/decade. (**b**) V_T_ and (**c**) I_SINW_ at different pH levels; these values were extracted from Fig. 2a. V_T_ was extracted using the constant current method at I_SINW_ = 10^−8^ A, and the current response (log (ΔI/I_0_)) was extracted from the transfer characteristics at V_LG_ = 0.65 V.

We first used the pH-sensing experiment as a model system to study the response of our hybrid SiNW and MOSFET biosensor. In these experiments, the SiO_2_ surface of the SiNWs were modified with 3-aminopropyltriethoxysilane (APTES). The SiNW surface was exposed to 0.1X potassium phosphate buffer solutions with five different pH values (*i.e*., pH 5, 6, 7, 8, and 9) by a microfluidic channel (see Supplementary Figure S2 online). In the pH experiment of a single SiNW FET with an electrolyte gate (*i.e*., a liquid gate), we used an n-type SiNW. The drain current flowing through the SiNW channel (I_SINW_) was measured at a constant drain voltage (V_D_) of 1 V while the liquid gate voltage (V_LG_) was swept from 0 to 1.2 V with 25 mV steps. The transfer characteristics of the n-type SiNW FET for the five different pH values are shown in Fig. 2a. The gate leakage current in this work is negligible (see Supplementary Figure S3 online). The pH responses are stable and repeatable over many hours of operations. As the pH level increases, the conductance of the n-type SiNW FET decreases, resulting in a positive shift of the threshold voltage (V_T_). This can be explained by the protonation/deprotonation of −NH_2_ and −SiOH groups on the functionalized SiNW surface. At low pH levels, the −NH_2_ group is protonated to −NH_3,_ resulting in a positive charge. In contrast, at high pH levels, the −SiOH group is deprotonated to −SiO^−^ and results in a negative charge^23^. The average V_T_ shift is 52 mV per pH (Fig. 2b), which is below the Nernst limit as expected. In addition, the SS of the single SiNW FET with the liquid gate is 147 mV/decade, which is considerably larger than that of the ideal value (59.6 mV/decade). In the subthreshold region, the extracted current response (how many decades per pH) of the single SiNW FET is approximately 1/3 decade per pH (Fig. 2c), which is smaller than the ideal current response of 1 decade per pH. Therefore, it is difficult for the single SiNW FET to achieve a higher current response because of the smaller V_T_ shift and the larger SS than the expected values, which is caused by non-ideal interfaces between the gate insulator layer and the electrolyte^23^.

**Figure 3:**
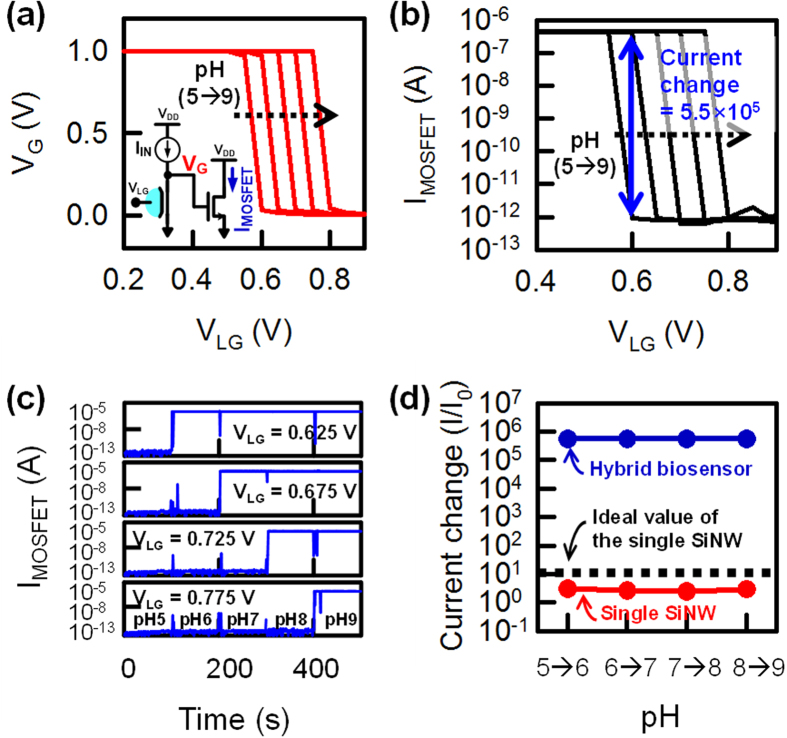
Measured electrical characteristics of the hybrid silicon nanowire (SiNW)-MOSFET biosensor as a pH sensor. (**a**) V_G_ of the hybrid biosensor while varying the pH from 5 to 9. (**b**) MOSFET output current (I_MOSFET_) as a function of pH level in the hybrid biosensor. The extracted current change is 5.5 × 10^5^ (=5.74 decade per pH). (**c**) Transient response of I_MOSFET_ while varying the pH in the hybrid biosensor. (**d**) Measured current change depending on the biosensor types, *i.e*., the single SiNW biosensor and the hybrid biosensor. The dashed black line indicates the maximum current response according to the Nernst limit.

Figure 3 presents the experimental results for the SiNW/MOSFET hybrid pair biosensor, demonstrating a high current response for pH changes. To understand the overall response of the biosensor, we first characterize the voltage transfer curves, V_LG_ versus V_G_, for different pH levels, as shown in Fig. 3a. In the measurement, the current source (I_IN_) is set to 1 nA, and the V_G_ node is limited to 1 V using the voltage compliance of the measurement equipment (4156C, Agilent). At low V_LG_ (*i.e*., the off-state of the SiNW FET), it is difficult to discharge the forced current I_IN_ into the ground through the SiNW channel because the conductance of the SiNW remains low. Thus, the V_G_ node maintains a ‘HIGH’ (=1 V) level. However, V_G_ rapidly changes from ‘HIGH’ to ‘LOW’ (=0 V) at a specific V_LG_ point because the conductance of the SiNW becomes sufficiently large (*i.e*., the on-state of the SiNW FET) to discharge I_IN_. Therefore, the rapid change of V_G_ is expected to be able to effectively change the output current (I_MOSFET_) in the MOSFET at the specific V_LG_ point.

Figure 3b shows the resulting output current (I_MOSFET_) of the MOSFET in our hybrid sensor. At low V_LG_, I_MOSFET_ remains high (5.5 × 10^−7^ A) because of the high V_G_ value at this point. However, I_MOSFET_ abruptly drops to a low level (10^−12^ A) at a specific V_LG_, as expected. As a result, we achieve a large current change (I/I_0_) with varying pH levels (approximately 5.5 × 10^5^/pH) at a fixed V_LG_ value (note the vertical arrow between pH curves in Fig. 3b). As shown in Fig. 3b, the current change of the hybrid biosensor is saturated, since this I/I_0_ is strongly affected by the on/off current ratio of the MOSFET (see Supplementary Figure S4 online); therefore, I/I_0_ can be further increased by improving the performance of the MOSFET.

Figure 3c shows I_MOSFET_ versus time with varying pH levels at the fixed V_LG_ value. Noise spikes occur every 100 s when the pH values are changed; however, the I_MOSFET_ quickly equilibrates. A clear amplified signal is obtained for the different pH levels by adjusting V_LG_, *i.e*., I/I_0_ ~ 5.5 × 10^5^, as shown in Fig. 3c. The hybrid biosensor gives a current response of 5.74 decade per pH, as compared to the small pH response of a single SiNW FET (a current response of 1/3 decade per pH). Finally, the extracted current response of the hybrid pH sensor and the conventional SiNW pH sensor alone are compared in Fig. 3d. The red symbols show the current response measured for the single SiNW FET as a function of pH. The current response is extracted from the transfer curves of the single SiNW biosensor in Fig. 2a at V_LG_ = 0.65 V. The dashed black line represents the aforementioned ideal current response of a single SiNW FET. The current response of the hybrid biosensor is represented by blue dots, demonstrating a current response approximately 2.5 × 10^5^ times greater than that of the single SiNW pH sensor. It is important to note that the hybrid biosensor has the wide dynamic range by shifting the liquid gate voltage (Fig. 3a). Importantly, we expect that a small threshold voltage shift (a change of 20 mV), corresponding to a 0.34 pH change, can be detected with the high current response of the hybrid biosensor, as shown in Supplementary Figure S5 online. This simulation also shows that we expect a smaller threshold voltage shift (less than ΔpH < 0.34) should be detectable (Supplementary Figures S6 and S7 online). Thus, the hybrid biosensor can simply produce a dramatic amplification of the current response without additional off-chip circuitry.

**Figure 4:**
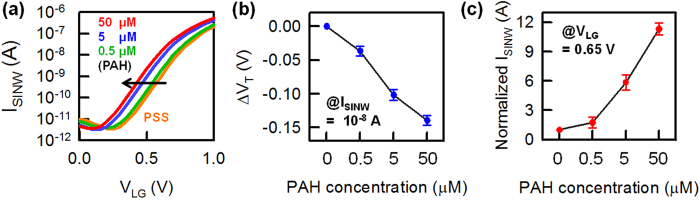
Measured electrical characteristics of the single SiNW FET as a function of PAH concentration. (**a**) Transfer curves of the single SiNW biosensor with increasing PAH concentration. (**b**) V_T_ shift and (**c**) normalized I_SINW_ are extracted from the transfer curves of the SiNW biosensors. The measured data from six devices are included in each condition.

**Figure 5:**
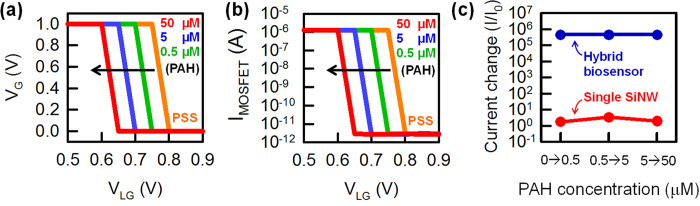
Measured electrical characteristics of the hybrid biosensor as a function of PAH concentration. (**a**) V_G_-V_LG_ and (**b**) I_MOSFET_-V_LG_ characteristics of the hybrid biosensor with varying PAH concentrations. (**c**) Comparison of the current change for the two biosensor types at different PAH concentrations.

To further evaluate the advantages of our hybrid sensor, we detected charged molecules, specifically poly(sodium styrene sulfonate) (PSS) and poly(allylamine hydrochloride) (PAH), which are oppositely charged. The PSS polymer is negatively charged, whereas the PAH polymer is positively charged, and layer-by-layer build-up is a straightforward protocol^24^,^25^,^26^. To detect the positive charges of the PAH polymer, the PSS polymers were pre-applied to the positively charged APTES-modified SiO_2_ surface. Next, the PAH polymer was attached on the PSS layer. The experimental procedure is explained in detail in Supplementary Figure S8 online. Figure 4 demonstrates the experimental validation of charged polymer detection using the single SiNW biosensor. The transfer curves of the n-type single SiNW FET with varying PAH concentrations are shown in Fig. 4a. The attachment of the positively charged PAH results in a negative V_T_ shift because the attached positively charged PAH can accumulate more electron carriers in the n-type SiNW channel. Thus, as the PAH concentration increases, the V_T_ of the n-type SiNW biosensor is more negatively shifted and the current flowing to the SiNW (*i.e.*, I_SINW_) increases correspondingly (Fig. 4b,c). In this case, the average ΔV_T_ is 38 mV at the lowest PAH concentration (500 nM), and the measured average current change is 2.7. Figure 5a shows that the V_G_-V_LG_ curves of the hybrid biosensor are negatively shifted with changing PAH concentration. The I_MOSFET_-V_LG_ curve is also shifted depending on the V_G_-V_LG_ curve shift. Therefore, similar to the pH experiment, the hybrid biosensor can obtain a large amplification in current change (=4.5 × 10^5^ at a PAH of 500 nM) compared with a single SiNW FET at a fixed V_LG_, as shown in Fig. 5b. The current change for two types of biosensors (*i.e*., a single SiNW and the hybrid sensor) at different PAH concentrations are summarized in Fig. 5c. The red symbols show the current response measured in the single SiNW biosensor with varying PAH concentrations at V_LG_ = 0.65 V. The blue symbols represent the current response of the hybrid biosensor. The experimental results demonstrate that the hybrid biosensor can dramatically increase the current response for charged polymer detection. The high current response of the hybrid biosensor as demonstrated here should be applicable to other biomolecule detection systems.

## Conclusions

In this paper, we demonstrated a hybrid biosensor composed of a single SiNW and a single amplifier MOSFET. We show that the hybrid biosensor offers remarkable amplification of the current response in both pH and charged polymer detection, which is more than 2.5 × 10^5^ times larger than a single SiNW sensor counterpart. Furthermore, the demonstrated biosensor shows a wide detection dynamic range by adjusting the liquid gate voltage. We believe that this is the first report of a high responsivity, large dynamic range amplifier monolithically integrated with a biosensor, which should be advantageous and practical for biosensor applications which requires lower noise, high speed, and high density. Given its compatibility with conventional top-down CMOS processing technology, this biosensor should have wide applicability for biomedical and chemical sensors.

## Additional Information

**How to cite this article**: Lee, J. *et al*. A Highly Responsive Silicon Nanowire/Amplifier MOSFET Hybrid Biosensor. *Sci. Rep*. **5**, 12286; doi: 10.1038/srep12286 (2015).

## Supplementary Material

Supplementary Information

## Figures and Tables

**Figure 1:**
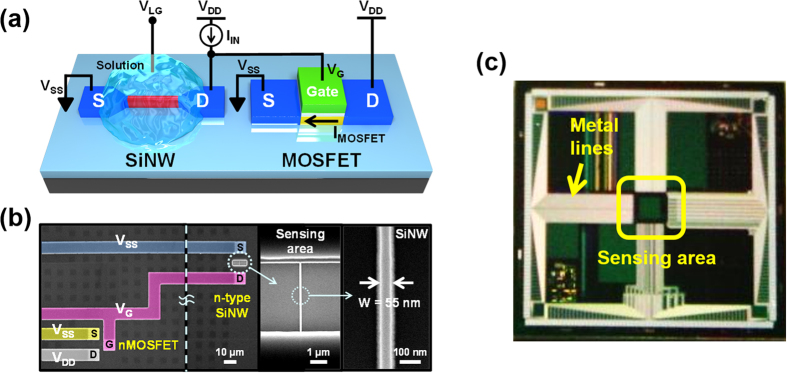
(**a**) Schematic diagram of the demonstrated biosensor composed of an n-type SiNW and a MOSFET. The SiNW is biased by V_LG_, but the MOSFET is isolated from the solution. (**b**) False-colored scanning electron microscope images of the biosensor. The insets show a magnified view of the SiNW channel region (W = 55 nm). (**c**) Photograph of the biosensor chip fabricated via a top-down method.

## References

[b1] Zheng G., Patolsky F., Cui Y., Wang W. U. & Lieber C. M. Multiplexed Electrical Detection of Cancer Markers with Nanowire Sensor Arrays. Nat. Biotechnol. 23, 1294–1301 (2005). 16170313 10.1038/nbt1138

[b2] Wang W. U., Chen C., Lin K.-h., Fang Y. & Lieber C. M. Label-free detection of small-molecule-protein interactions by using nanowire nanosensors. Proc. Natl. Acad. Sci. USA 102, 3208–3212 (2005). 15716362 10.1073/pnas.0406368102 PMC548960

[b3] Bergveld P. Development, Operation, and Application of the Ion-Sensitive Field-Effect Transistor as a Tool for Electrophysiology. IEEE Trans. Biomed. Eng. 19, 342–351 (1972). 5038390 10.1109/TBME.1972.324137

[b4] Stern E. . Label-free immunodetection with CMOS-compatible semiconducting nanowires. Nature 445, 519–522 (2007). 17268465 10.1038/nature05498

[b5] Patolsky F. & Lieber C. M. Nanowire nanosensors. Mater. Today 8, 20–28 (2005).

[b6] Ramachandran N., Larson D. N., Stark P. R. H., Hainsworth E. & LaBaer J. Emerging tools for real-time label-free detection of interactions on functional protein microarrays. FEBS J. 272, 5412–5425 (2005). 16262683 10.1111/j.1742-4658.2005.04971.x

[b7] Cheng Y. . Mechanism and Optimization of pH Sensing Using SnO_2_ Nanobelt Field Effect Transistors. Nano Lett. 8, 1479–4184 (2008). 10.1021/nl801696b PMC2771949 19367840

[b8] Scanrano S., Mascini M., Turner A. P. F. & Minunni M. Surface plasmon resonance imaging for affinity-based biosensors. Biosens. Bioelect. 25, 957–966 (2010). 10.1016/j.bios.2009.08.039 19765967

[b9] Buchapudi K. R., Huang X., Yang X., Ji H.-F. & Thundat T. Microcantilever biosensors for chemicals and bioorganisms. Analyst 136, 1539–1556 (2011). 21394347 10.1039/c0an01007c

[b10] Bruno A. E. . All-Solid-State Miniaturized Fluorescence Sensor Array for the Determination of Critical Gases and Electrolytes in Blood. Anal. Chem. 69, 507–513 (1997). 9030059 10.1021/ac960855n

[b11] Cui Y., Wei Q., Park H. & Lieber C. M. Nanowire Nanosensors for Highly Sensitive and Selective Detection of Biological and Chemical Species. Science 293, 1289–1292 (2001). 11509722 10.1126/science.1062711

[b12] Ahn J.-H. . Double-Gate Nanowire Field Effect Transistor for a biosensor. Nano Lett. 10, 2934–2938 (2010). 20698606 10.1021/nl1010965

[b13] Gao A. . Enhanced Sensing of Nucleic Acids with Silicon Nanowire Field Effect Transistor Biosensors. Nano Lett. 12, 5262–5268 (2012). 22985088 10.1021/nl302476h

[b14] Lee J. . A Novel SiNW/CMOS Hybrid Biosensor for High Sensitivity/Low Noise. 59th IEEE International Electron Device Meeting (IEDM), Washington DC, USA, 385–388 (2013).

[b15] Park I., Li Z., Pisano A. P. & Williams R. S. Top-down fabricated silicon nanowire sensors for real-time chemical detection. Nanotechnol. 21, 015501-1–015501-9 (2010). 10.1088/0957-4484/21/1/015501 19946164

[b16] Curreli M., Thompson M. E. . Real-Time, Label-Free Detection of Biological Entities Using Nanowire-Based FETs. IEEE Trans. Nanotechnol. 7, 651–667 (2008).

[b17] Elfström N. . Surface Charge Sensitivity of Silicon Nanowires: Size Dependence. Nano Lett. 7, 2608–2612 (2007). 17691849 10.1021/nl0709017

[b18] Nair P. R. & Alam M. A. Design Considerations of Silicon Nanowire Biosensors. IEEE Trans. Elect. Dev. 54, 3400–3408 (2007).

[b19] Rajan N. K., Routenberg D. A. & Reed M. A. Optimal signal-to-noise ratio for silicon nanowire biochemical sensors. Appl. Phys. Lett. 98, 264107 (2011). 21799538 10.1063/1.3608155 PMC3144966

[b20] Gao X. P. A., Zheng G. & Lieber C. M. Subthreshold Regime has the Optimal Sensitivity for Nanowire FET Biosensors. Nano Lett. 10, 547–552 (2010). 19908823 10.1021/nl9034219 PMC2820132

[b21] Lu M.-P., Hsiao C.-Y., Lai W.-T. & Yang Y.-S. Probing the sensitivity of nanowire-based biosensors using liquid-gating. Nanotechnol. 21, 425505-1–205505–425505-5 (2010). 10.1088/0957-4484/21/42/425505 20864778

[b22] Taur Y. & Ning T. H. Fundamentals of Modern VLSI Devices, Cambridge University Press, Cambridge, 1998.

[b23] Bousse L., Rooij N. F. D. & Bergveld P. Operation of chemically sensitive field-effect sensors as a function of the insulator-electrolyte interface. IEEE Trans. Elect. Dev. ED-30, 1263–1270 (1983).

[b24] Riegler H. & Essler F. Polyelectrolytes. 2. Intrinsic or Extrinsic Charge Compensation? Qunatitative Charge Analysis of PAH/PSS Multilayers. Langmuir 18, 6694–6698 (2002).

[b25] Uslu F. . Labelfree fully electronic nucleic acid detection system based on a field-effect transistor device. Biosens. Bioelect. 19, 1723–1731 (2004). 10.1016/j.bios.2004.01.019 15142607

[b26] Vu X. T., Stockmann R., Wolfrum B., Offenhäusser A. & Ingebrandt S. Fabrication and application of a microfluidic-embedded silicon nanowire biosensor chip. Phys. Status Solidi A 207, 850–857 (2010).

