# Supplementary material for: A Highly Responsive Silicon Nanowire/Amplifier MOSFET Hybrid Biosensor
Source: Sci Rep. 2015 Jul 21;5:12286. doi: 10.1038/srep12286 (PMC4508832; doi:10.1038/srep12286)
Supplement: Supplementary Information [file srep12286-s1.doc]

A Highly Responsive Silicon Nanowire/Amplifier MOSFET Hybrid Biosensor

Jieun Lee^1.2^, Jaeman Jang^1^, Bongsik Choi^1^, Jinsu Yoon^1^, Jee-Yeon Kim^3^, Yang-Kyu Choi^3^, Dong Myong Kim^1^, Dae Hwan Kim^1^, and Sung-Jin Choi^1,*^

^1^School of Electrical Engineering, Kookmin University, Seoul 136-702, Republic of Korea

^2^Department of Electrical Engineering, Yale University, New Haven, Connecticut 06511, United States

^3^Department of Electrical Engineering, Korea Advanced Institute of Science and Technology, Daejeon 305-701, Korea

**Corresponding Author**

^*^E-mail: [sjchoiee@kookmin.ac.kr](mailto:sjchoiee@kookmin.ac.kr)

**Figure S1. Schematic of the fabrication procedure for integrating the SiNW with the MOSFET**

The hybrid biosensors were fabricated on a boron-doped (4×10^15^ cm^-3^) 6” (100) silicon-on-insulator (SOI) wafer, and the top 100nm-thick Si layer was separated from the Si substrate by a 375nm-thick buried oxide (BOX). Details of the fabrication procedure are provided in Figure S1.

To build the buffer oxide as protection for implantation, a 20nm-thick SiO_2_ layer was formed on top of the Si layer through dry oxidation. As a result, the thickness of the top Si layer was reduced to 90 nm. Subsequently, channel implantation was conducted for the p-type region (B^+^, energy: 20 keV, dose: 5×10^13^ cm^‑2^) and the n-type region (P^+^, energy: 40 keV, dose: 3×10^13^ cm^‑2^). After stripping the buffer oxide in HF solution, the annealing process was conducted at 950°C for 30 min. Then, the device active region was patterned on the Si layer by the mix-and-match process of e-beam lithography combined with conventional photolithography. By using the hydrogen silsesquioxane (HSQ) and photoresist (PR) as an etch mask, the 90nm-thick Si layer was anisotropically etched in the HBr/O_2_-based inductively coupled plasma (ICP) chamber. Moreover, a 10nm-thick gate oxide layer grows on the active region with dry oxidation at 850°C; thus, the SiNW width is reduced. Next, an additional 10nm-thick oxide was deposited at 780°C with a low-pressure chemical vapor deposition (LPCVD) system. This 20nm-thick SiO_2_ layer becomes the gate dielectric of the MOSFET part. After complete formation of the gate oxide, the 100nm-thick poly-Si was deposited at 630°C via an LPCVD system to form the poly-Si top gate in the MOSFET. It was patterned by the conventional photolithography and formed by an ICP etch system. The gate oxide layer protects the SiNW during the etching of the poly-Si layer. The poly-Si top gate of the MOSFET was patterned by conventional photolithography and formed by the ICP etch method. By using a PR mask, which covers only the SiNW channel, the source/drain regions of the SiNW were doped with As^+^ ion implantation for an n-type source/drain. The source, drain, and top gate of the MOSFET device were also doped simultaneously. After PR stripping, the annealing step followed at 1,000°C for 30 min for the dopant activation. Then, 500nm-oxide was deposited as an inter-layer dielectric via high-density plasma (HDP) chemical vapor deposition (CVD), followed by the planarization process through chemical mechanical planarization (CMP). After the CMP process, the contact hole was formed by photolithography and magnetic-enhanced reactive ion etching (MERIE) in CHF_3_/CF_4_ plasma. After metallization using Al sputtering, photolithography, and MERIE in BCl_2_/Cl_2_ plasma, tetraethyl orthosilicate (TEOS) was deposited as a passivation oxide to protect the MOSFETs and metal lines. For electrical measurement, the oxide layer on the metal pad region was etched by photolithography and MERIE in CHF_3_/CF_4_ plasma. Finally, the sensing area on the SiNW channel region became open and connected with the SiNW surface and bio-molecule analyte by removing the TEOS passivation oxide via MERIE in CHF_3_/CF_4_ plasma. The completion of the alloy process represents the end of the entire process.

**Figure S2. PDMS fluidic channel. (a) Fabricated PDMS channel master on a 4” Si wafer. (b) Top view and (c) bird’s eye view of the PDMS on the chip. (d) Measurement setup with tubing lines on the probe station.**

A polydimethylsiloxane (PDMS) fluidic channel was prepared for fluidic transport of the solution. First, the PDMS master was fabricated on a 1mm-thick 4” Si wafer in Figure S2a. The master was filled with a fully stirred mixture of 40 g of Sylgard-184 silicone elastomer and 4 g of Sylgard-184 silicon elastomer curing agent and was then baked at 80°C for 3 h. Next, the cured PDMS was taken off and cut to fit the final chip dimensions around the channel. The volume of the cell is as follows: the size of the fluidic channel on the chip is approximately 4.6 mm × 3 mm × 200 µm (length × width × height). To construct a PDMS fluidic channel on the chip (Figure S2b and c), the PDMS fluidic channel and chip were exposed to UV O_3_ for 330 s and bonded together. Then, the combined element was heated on a hotplate at 120°C for 10 min. The final measurement setup with tubing lines (inner diameter=750 µm, outer diameter=1.6 mm) on a probe station is shown in Figure S2d.

**Figure S3. Comparison of measured I_SINW_ with I_LG_ of the SiNW biosensor (@ V_D_ = 1V).**

The measured liquid gate leakage current (I_LG_) compared with I_SINW_ is shown in Figure S3. The leakage is insignificant, of order a few pico amps (~ 4 orders of magnitude less than the channel current).

**Figure S4. Measured (a) transfer and (b) output characteristics of the fabricated SOI nMOSFET (W/L=2/2 μm) of the hybrid biosensor.**

The measured transfer and output characteristics of the fabricated SOI nMOSFET are shown in Figures S4a and S4b, respectively. The current of the nMOSFET (I_MOSFET_) is well modulated by the gate and drain biases (V_G_ and V_D_). The current on/off ratio (I_ON_/I_OFF_) of the MOSFET is extracted from I_ON_=I_MOSFET_ (@ V_G_=V_D_=1 V) and I_OFF_=I_MOSFET_ (@ V_G_=0 V, V_D_=1 V).

**Figure S5. (a) Applied square wave of V_LG_ with a period of 20 s. (b) Measured current of both the single SiNW biosensor (red line) and hybrid biosensor (blue line) with V_LG0_=0.65 V and V_p-p_=20 mV.**

Figure S5 shows the minimum detectable signal amplitude of the hybrid biosensor. We applied the 20mV peak-to-peak (V_p-p_) square wave to the V_LG_ with a period of 20 s, as shown in Figure S5a. As a result, the current change is 1.39 for the single SiNW biosensor and 5×10^5^ for the hybrid biosensor, as shown in Figure S5b. The results demonstrate that the hybrid biosensor can detect a small bio-signal when its voltage signal amplitude is 20 mV. Thus, the high current change can detect at least Δ0.34 pH, which corresponds to a 20 mV surface potential change.

To back up the claim of the derived pH sensitivity of 0.34, we also performed a SPICE simulation using the calibrated circuit models for comprehensive theoretical analysis (Figure S6, showing the measured versus simulated I-V characteristics of the SiNW and nMOSFET devices), shown below. This simulation also shows that we expect a smaller threshold voltage shift (less than ΔpH < 0.34) should be detectable (Figure S7).

**Figure S6. Comparison of measured with simulated I-V characteristics of the SiNW and nMOSFET**

**Figure S7. Comparison and expectation of the current change in the biosensors. We assumed that the applied V_p-p_ corresponds to the threshold voltage shift of the SiNW biosensor caused by the pH changes. Symbol and line data show the experimental and simulated results, respectively.**

**Figure S8. Schematic of the surface treatment procedure using the charged polymer (negatively charged PSS and positively charged PAH).**

Figure S8 shows a schematic of the functionalization method for charged polymer detection. The devices were treated by UV O_3_ for 330 s to introduce more than the standard number of hydroxyl (-OH) groups to the Si oxide surface. Then, the device surfaces were silanized with 1% 3-aminopropyltriethoxysilane (APTES) in 95% ethanol solution for 30 min. After rinsing with 95% ethanol and blowing dry with N_2_ three times, the devices were functionalized with 50μM PSS in distilled water (DW) for 1 h. Next, the devices were rinsed three times with DW and blown dry with N_2_. Finally, the devices were covered with PAH in DW at different concentrations (0.5-50 μM). All measurements related to the charged polymer were conducted in 1X phosphate buffered saline (PBS) solution.
